# Self-medication with analgesics among medical students and interns in King Abdulaziz University, Jeddah, Saudi Arabia

**DOI:** 10.12669/pjms.311.6526

**Published:** 2015

**Authors:** Nahla Khamis Ibrahim, Banan Mohammad Alamoudi, Wejdan Omar Baamer, Rajaa Mohammad Al-Raddadi

**Affiliations:** 1 Prof. Nahla Khamis Ibrahim, MBBS, MPH, DHPE, PhD.Family and Community Medicine Department, King Abdulaziz University, Jeddah, Saudi Arabia. Epidemiology Dept., High Institute of Public Health,Alexandria University, Alexandria, Egypt.; 2 Dr. Banan Mohammad Alamoudi, MBBS. Family and Community Medicine Department, King Abdulaziz University, Jeddah, Saudi Arabia.; 3 Dr. Wejdan Omar Baamer, MBBS. Intern, King Abdulaziz University, Jeddah, Saudi Arabia.; 4 Dr. Rajaa Mohammad Al-Raddadi, MBBS, PhD. Consultant Community Medicine, Ministry of Health, Jeddah, Saudi Arabia.

**Keywords:** Analgesics, Self-medication, Medical students

## Abstract

**Objectives:**

To determine the prevalence and predictors of self-medication with analgesics among senior medical students and interns in King Abdulaziz University (KAU), Jeddah, Saudi Arabia.

**Methods:**

A cross-sectional study was conducted among 504 participants in 2013. A multistage stratified random sampling was used. A confidential, anonymous & self-administered questionnaire was used to collect personal & socio-demographic data. Data about self-medication and self-medication with analgesics during the preceding 6 months were also inquired. Both descriptive and analytical statistics were done by SPSS version 18 & Epi-Info.

**Results:**

During the 6 months preceding the study, 75.2% and 55.4% of participants used self -medication & analgesic self-medication, respectively. The first predictor of utilization of analgesic self-medication was living with family (aOR; 1.96, 95% CI: 1.22-3.14), followed by age >21 years & non- professional jobs of fathers.

**Conclusion:**

Alarming high rates of self medication and self-medication with analgesics were observed among medical students and interns. Self-medication needs improvement through educational, regulatory and managerial strategies.

## INTRODUCTION

Self-medication with Over-the-Counter (OTC) drugs is a worldwide public health problem^1^ which is more experienced in developing countries.^2^ Self-medication can be defined as “the use of drugs to treat self-diagnosed disorders or symptoms, or the intermittent or continued use of a prescribed drug for chronic or recurrent disease or symptoms”.^3^ Studies revealed that self-medication represents a common problem among university students.^4^^-^^6^

It was also illustrated that the increase of OTC medication was influenced by many factors like age, sex, medication knowledge, previous experience of disease, non-seriousness of illness.^5^^,^^7^^-^^9^ Medications usually obtained from home as a left-over, from pharmacies and from family members.^10^^,^^11^

Self-medication increases the chance of illegal drug use, dependence and masking the underlying disease which lead to public health complication, generate drug resistance and impede diagnosis.^12^ Governments and health authorities have to make sure that self-medication is performed in a responsible manner, and lay the foundation of that only safe drugs are made available OTC and that patients are given sufficient information regarding drug use, their contraindication and side effects.^13^

Self-medication in Saudi Arabia seems to be a common practice among the general population.^14^ Young adults especially the students usually made unprotected health related decisions that may affect their health.^15^ Studies conducted among university students worldwide has reported that the utilization of OTC drugs among university students is common and inappropriate.^10^^,^^16^^-^^19^ The use of analgesics is widespread worldwide. Some analgesics are categorized as OTC drugs and some are not. Side-effects experienced may vary with different people, and overuse could be harmful.^1^ However, to the best of our knowledge little studies were conducted regarding analgesic self-medication, especially in Jeddah. So, such study is needed. The objectives of the current study were to determine the prevalence and predictors of self-medication and analgesic self-medication among senior medical students and interns in King Abdulaziz University (KAU).

## METHODS

***Ethical statement:*** The study was approved by the Institutional Review Board (IRB) of KAU. It was conformed to the ethical standards of the Helsinki declaration. 

A cross-sectional sampling was conducted among clinical years senior clinical years medical students (4^th^ - 6^th^ year) and interns in KAU, Jeddah, 2013. The Sample size was determined by the formulae:

$n=\frac{z^{2}\times p\times q}{d^{2}}$

Where *n*: the minimum sample size, *Z *= constant (1.96) and *p* is the prevalence of self-medication assumed to be 0.73% on the basis of a previous study done in Bahrain^20^ and *q = (1-p) =*0.27. The minimum calculated sample size to achieve a precision of ± 4% with a 95% Confidence Interval (CI) was 473. For stratification purposes, the sample size was increased to 504 during the fieldwork. A multistage stratified random sampling technique was used. Stratification took into consideration the students' gender and the educational year. 

Data were collected using a validated, constructed, anonymous, confidential and self-administered questionnaire. Reliability of the questionnaire was evaluated using Cronbach’s α test and it was found to be 85%. The questionnaire consisted of closed ended questions and inquired about personal and socio-demographic data, use of self-medication and self-medication with analgesics during the six months preceded the study. Statistical analyses were performed by Statistical Package for Social Sciences version 18 (SPSS Inc., Chicago, Ill., USA) & Epi-Info programs. Both descriptive and analytic statistics were done.

## RESULTS

The total sample amounted to 504 participants with a mean of 22.9 ± 1.2 years. Male to female ratio was almost equal (1.08: 1). The majority of participants were Saudi (92%) and single (89.1%). Most of the students & interns lived with their families (81.5%) and with enough family income (98.6%). 

About three-fourths (75.2%) of participants reported using self-medications during the 6 months preceded the study. Table-I shows that analgesics were the most frequently (55.4%) self-medication used by medical students and interns. This is followed by antipyretics (29.0%), antihistaminic (27.0%) and antibiotics (25.8%).

Table-II reveals that participants aged > 21 years had a significantly higher prevalence of using analgesic self-medication compared to others (*X*^2^= 6.35, *p *<0.01). The prevalence of use was also increased by increasing academic year with the highest level among the sixth year students (OR= 2.15, 95% CI: 1.31-3.56). On the other hand, there is no statistical significant difference between gender and use of analgesic self-medication.

Predictors of utilization of analgesic self-medication, after controlling confounding factors is presented in Table-III. Students living with their families & those aged > 21 years had about twice the risk of using analgesic self medication compared to others. Students who had non-professional working fathers had about 1.5 risk of utilizing analgesic self-medication (aOR =1.55; 95% CI: 1.01-2.38)

Study respondents reported that the most common symptoms for using self-medication with analgesics were headache (33.6%), followed by common cold (17.5%), dysmenorrhea (13.8%) and bone and joints pain (5.3%). 

The study showed that among participants who used analgesic self medication, Non Steroidal Anti-inflammatory Drugs (NSAIDs) were utilized by 49.6% and 47.2% used acetaminophens. The majority (86.7%) of participants who used self-medication with analgesics didn’t encounter side effects while 13.3% reported side effects. 

The causes which lead participants to use analgesic self-medication were that the problem was not serious (35.4%), previous experience with the same drug (27.2%), lack of time (15.1%), urgency of problem (12.1%), cost of consultation (4.3%), advice from friend (3.9%) and unavailability of transportation (2.0%).

## DISCUSSION

The present study revealed high prevalence (75.2%) of self-medication by medical students and interns in KAU. This agrees with results from an Indian study done among first and third year medical students (76.3%).^21^ On the other hand, these rates are much higher than rate reported among population aged ≥ 16 years from Spain (12.7%).^22^ This discrepancy may be attributed to differences in the duration of reporting of self-medication, sample size, target populations or the level of knowledge about self-medication.

Our study showed that the prevalence of self-medication with analgesics was 55.4%. Some higher rates were reported among university students from Iran,^1^ from Egypt^17^ and among 1^st^ year medical students from Bahrain.^20^ These higher rates may be attributed to differences in sample size, gender selection or the type of the target populations.

On the other hand, a lower rate (38.8%) was reported among undergraduate nursing students from Brazil. This variation could be due to the differences in the study population. As the majority of the Brazilian were females. This may be also attributed to success of the Brazilian Health Surveillance Agency which has some regulations regarding monitoring advertisement which may help prevent problems such as self-medication.^23^

The present study showed that the prevalence of use of analgesic self-medication was higher among participants aged > 21 years compared to others. This might be because older students feel that they are able to self-medicate themselves with analgesics as they studied pharmacology. This result goes in line with results of Awad, et al.^9^

In the current study, students of the senior academic years (6^th^ year) used self-medication more than juniors. Other studies also reported the same results which are most probably due to increase in the level of medical knowledge and public health clinical training.^4^^,^^7^^,^^9^^,^^20^^,^^24^ On the other hand, some other studies showed no relationship between academic year and self-medication.^6^^,^^16^

Students who lived with their families used analgesic self-medication more often than students lived in dormitory. On the other hand, the Spanish study^22^ showed that self-medication was more prevalent among persons who lived alone. The reason of this difference could be because students lived with their families in the current study may be influenced by their parents knowledge, attitude and practice regarding self-medication compared to students lived alone. In contrast to the previously reported studies,^9^^,^^17^^,^^19^^-^^22^ we did not find any significant difference between gender and the use of analgesic self-medication.

Regarding the types of analgesics used NSAIDs and acetaminophen were the most common types of analgesics used by the students. This coincides with results of the previous studies.^1^^,^^5^^,^^18^ Headache followed by common cold were the most commonly reported complaints in the 6 months period prior to the study, which is similar to that mentioned by 1^st^ year medical students in Bahrain^19^ and university students of Brazil.^16^

The most common (35.4%) cause of use of analgesic self-medication in the current study was the non- serious problem which agrees with other studies.^5^^,^^20^^,^^25^ The study of Awad, et al.^9^ reported that the participants used pharmacies because they are of a lower cost compared to other health care facilities. Students from Brazil^23^ and India^24^ perceived that time saving were the most reason for using self-medication. These results also coincide with our results.

**Table-I:** Therapeutic classes of self-medication used by medical students and interns in King Abdulaziz University

*Drug*	*Frequency*	*Percent*
Analgesics	279	55.4
Antipyretics	146	29.0
Anti-histaminic	136	27.0
Antibiotics	130	25.8
Nutritional supplements	106	21.0
Anti-inflammatory	88	17.5
Antacids	77	15.3
Others	106	21.0

N.B. Each question was separately asked (mutually exclusive)

**Table-II:** Relationship between personal, socioeconomic factors and utilization of analgesic self medication among medical students and interns in King Abdulaziz University

*Variables*	*Self-medication with Analgesics in the past 6 months*				*Total*	*X2*	*OR*
	*Yes*		*No*			*(P)*	*(95%CI)*
	*No.*	*%*	*No.*	*%*			
*Age*							
≤ 21 years	33	42.3	45	57.7	78	6.35	0.53
> 21 years	246	57.7	180	42.3	426	(0.012)	(0.32-0.87)
*Sex*							
Male	132	54.5	110	45.5	242	0.12	0.93
Female	147	56.1	115	43.9	262	(0.72)	(0.66-1.33)
*Nationality*							
Saudi	261	55.9	206	44.1	467	0.98	1.43
Non Saudi	15	46.9	17	53.1	32	(0.32)	(0.7-2.94)
*Marital status*							
Single	248	55.4	200	44.6	448	0.02	0.96
Non-single	31	56.4	24	43.6	55	(0.88)	(0.54-1.68)
*Educational year*							
Fourth (RC)	55	43.0	73	57.0	128	11.53	1.71 (1.01- 2.99)
Fifth	71	56.3	55	43.7	126	(0.009)	2.15 (1.31-3.56)
Sixth	99	61.9	61	38.1	160		1.99 (1.11-3.58)
intern	54	60.0	36	40.0	90		
*Residual status*							
With family	239	58.2	172	41.8	411	7.03	1.84
In school or private dormitory	40	43.0	53	57.0	93	(0.008)	(1.16-2.90)
*Father's education*							
University and above	203	55.3	164	44.7	367	0.00	1.0
Less than university	73	55.3	59	44.7	132	(0.99)	(0.67-1.49)
*Mother's education*							
University and above	172	55.7	137	44.3	309	0.01	1.02
Less than university	107	55.2	87	44.8	194	(0.91)	(0.71-1.46)
*Father occupation*							
Professional	197	53.2	173	46.8	370	4.12	0.64
Non-professional	79	63.7	45	36.3	124	(0.042)	(0.42-0.98)
*Mother occupation*							
Professional	141	58.8	99	41.3	240	1.77	1.27
Non-professional	133	52.8	119	47.2	252	(0.18)	(0.89-1.82)
*Health insurance*							
Yes	103	53.6	89	46.4	192	0.37 (0.54)	0.89
No	171	56.4	132	43.6	303		0.62-1.28)
*Family Income:*							
Sufficient	274	55.4	221	44.6	495	Fisher’s 0.70	1.65
Non-sufficient	3	42.9	4	57.1	7	(0.38)	(0.36-7.46)

^(RC^^)^: Referent category

**Table-III:** Predictors of utilization of self-medication with analgesics among medical students and interns in King Abdulaziz University

*Variable*	*B*	*P*	*OR*	*95% CI*
Living with family	0.675	0.005	1.964	1.22 – 3.14
Age ( > 21 old)	0.667	0.009	1.949	1.18 – 3.22
Non-professional father job	0.443	0.042	1.557	1.01 – 2.38
Constant	- 0.798	0.000	0.45	

## Authors’ Contribution:

**NKI: **Design, write, revised, editing and final approval of manuscript.

**BMA: **Design, did data collection and analysis, and manuscript writing.

**WOB: **Did data collection, data entry, helped in writing.

**RMA: **Design, did review of manuscript

## CONCLUSION

High prevalence of self-medication and self-medication with analgesic prevails among medical students and interns in KAU. Older students, higher educational year, non-professional working fathers and living with families are predictors of analgesic self-medication use. Drug utilization among young adults needs improvement through educational, regulatory and managerial strategies. Researchers, public health practitioner and decision makers need to establish educational programs on analgesic self-medication. There is an urgent need to develop strict policies and legislations to prohibit the supply of drugs without prescription except the drugs that can be utilized safely as OTC. More studies are needed among general population to explore the various factors affecting self-medication. 

## Notes

***Grant Support & Financial Disclo***sures: None.
